# The protective role of whole grains and legumes against gastric cancer

**DOI:** 10.1515/biol-2025-1303

**Published:** 2026-05-04

**Authors:** Sarrah Daban, Manar Adawi, Dania Alsayyad, Rana Youssef, Sabika Allehdan, Aya Hamdan, Reema Tayyem

**Affiliations:** Department of Nutrition Sciences, College of Health Sciences, QU Health, Qatar University, PO Box 2713, Doha, Qatar; Allied Health Sciences Department, College of Health and Sport Sciences, University of Bahrain, P.O. Box 32038, Manama, kingdom of Bahrain

**Keywords:** whole grains, legumes, pulses, gastric cancer

## Abstract

Despite significant advancement in medicine, survival rates for gastric cancer (GC) remain low, especially when diagnosis occurs at advanced stages. Therefore, prevention through lifestyle and dietary modification has become a critical strategy to reduce the burden of GC. This review explores the relationship between the consumption of whole-grains (WG) and legumes and the risk of GC, and highlights the biological mechanisms that may contribute to their protective effects. A comprehensive search was conducted in PubMed/MEDLINE, Embase, ProQuest, Google Scholar, Web of Science, and Scopus for English-language studies published between 1996 and 2025. The search terms included gastric cancer, whole grains, legumes, beans, lentils, soy beans, and peanuts. Observational studies assessing WG and legumes intake in relation to GC risk were reviewed. Observational studies show controversial findings; the majority indicate that the consumption of WG and legumes is associated with lower risk of GC. WG are rich in fiber, antioxidants, vitamins, minerals, and phytochemicals as compared with refined grains, which help mitigate oxidative stress, inflammation, and DNA damage – key pathways involved in gastric carcinogenesis. Similarly, legumes contain beneficial bioactive compounds such as polyphenols, flavones, and isoflavones that may inhibit tumor initiation and slow progression. Incorporating WG and legumes into daily diets may represent an effective, sustainable, and accessible public health approach to lowering GC risk, while also supporting metabolic, immune, and gut health.

## 1 Introduction

Gastric cancer (GC) arises from the inner lining of the stomach and comprises tumors classified by histopathology, with adenocarcinoma being the most common diagnosis [1]. Approximately 90–95 % of GC cases originate in the gastric mucosa and are adenocarcinomas, while the remaining 5–10 % include malignant stromal tumors, gastric lymphomas, and other types arising from distinct gastric cell types [2], 3].

GC remains a major global health concern. In 2022, the World Health Organization (WHO) and the Global Cancer Observatory (GLOBOCAN) estimated it to be the fifth most common cancer worldwide, with nearly 970,000 new cases and more than 660,000 deaths across both genders. Asia bore the highest burden, accounting for over 70 % of incident cases and deaths, followed by Europe (14 % of cases, 14.5 % of deaths) and Latin America and the Caribbean (7.7 % of cases, 8.8 % of deaths). Africa, North America, and Oceania together contributed less than 7 % of the global incidence. Within the Eastern Mediterranean Region (EMRO), which includes Arab countries, Afghanistan, Iran, and Pakistan, approximately 37,000 new cases represented 3.9 % of the global total. Incidence varied across EMRO countries, ranging from higher rates in Iran and Afghanistan to lower rates in Qatar, Lebanon, Kuwait, and Sudan [1], 2].

Without early treatment, GC is universally lethal. Surgery remains the cornerstone of management because many GC types show relative resistance to chemotherapy and radiotherapy. Despite advances in diagnosis and treatment, GC continues to rank among the leading causes of cancer-related mortality worldwide [3]. Dietary habits and social behaviors substantially influence cancer risk and are estimated to account for roughly 50 % of GC cases [4]. Conversely, diets rich in fresh fruits and vegetables, WG, and healthy fats are associated with reduced GC risk and therefore represent promising targets for prevention [5], [6], [7]. A systematic review and meta-analysis encompassing 11 observational studies and about one million participants reported that adherence to the Mediterranean diet (MD), which is abundant in fruits, vegetables, grains, legumes, nuts, olive oil, and fish, may provide protection against gastric cancer (GC) and its various subtypes [8]. This protective effect is primarily due to the diet’s high levels of folate, polyphenols, and vitamins C and E, which combat oxidative stress and inflammation, helping to prevent DNA damage, abnormal cell growth, angiogenesis, and metastasis [9]. Additionally, a multisite case–control study revealed that individuals in the highest tertile of legumes consumption had a significantly reduced risk of GC compared to those with the lowest tertile [10]. Legumes are rich in dietary fiber, protein, vitamin E, B-vitamins, selenium, isoflavones, and lignans, all of which may have cancer-preventive properties [10].

In practice, the number of whole grains (WG) and legumes consumed is influenced by various contextual factors. Socioeconomic aspects such as price, availability, and labeling, along with cultural preferences (for instance, a preference for refined rice or white bread and the customary use of salted or fermented soybean pastes), and geographic elements (including local agricultural practices and soil micronutrient content) determine whether individuals can realistically replace refined staples or processed meats with WG and legumes. These factors clarify why associations vary among countries: in regions where WG foods are costly or scarce, or where legumes are primarily consumed as salty condiments, the potential for risk reduction is limited; whereas, in areas where affordable WG products and minimally salted legumes are widely available, the potential benefits are greater.

Given this context, the present narrative review analyzes existing research on the relationship between the consumption of WG and legumes and the risk of GC. It also synthesizes current evidence regarding the potential effects of consuming WG and legumes and explores the mechanisms through which they may influence the development of GC.

## 2 Methodology

A comprehensive search was conducted using multiple databases, including PubMed/MEDLINE, ProQuest, Embase, Google Scholar, Web of Science, and Scopus to identify relevant publications from 1996 to 2025. The search strategy employed terms such as “gastric cancer,” “stomach cancer,” “stomach adenocarcinoma,” “grains,” “whole grains,” “legumes,” “beans,” “lentils,” “soybeans”, and “peanuts.” The narrative review included observational studies such as case-control studies, cohort studies and systematic reviews and meta-analyses, which examined the association between GC and the consumption of WG and legumes across different regions. The inclusion criteria were restricted to studies relevant to the review’s objectives and published in English. A summary of the search strategy is provided in Table 1.

**Table 1:** Summary of the search approach used in this literature review.

Items	Details
Databases and other sources searched	PubMed/MEDLINE, ProQuest, Embase, Google Scholar, Web of Science, and Scopus
Search terms used	gastric cancer,” “stomach cancer,” “stomach adenocarcinoma,” “grains,” “whole grains,” “legumes,” “beans,” “lentils,” “soybeans,” and “peanuts”
Time frame	1996–2025
Inclusion/exclusion criteria	Observational studies such as case-control studies, cohort studies, and systematic reviews and meta-analyses. Only English-language studies were considered
Collection procedure	Literature search was conducted by all authors independently

## 3 Whole grains intake and GC

Grains are essential components of the human diet, contributing significantly to global energy and nutrient intake while forming the largest part of the recommended daily allowance [11]. The American Association of Cereal Chemists (AACC) defines whole grains (WG) as “the intact, ground, cracked, or flaked caryopsis, where the principal anatomical components – the starchy endosperm, germ, and bran – are present in the same relative proportions as found in the intact caryopsis.” [12] Grains are categorized as either whole grains or refined grains based on processing methods such as cleaning, hulling, milling, parboiling, and drying [13]. Refinement typically removes the bran and germ. In comparison to refined grains, whole grains are higher in dietary fiber, B vitamins, vitamin E, iron, magnesium, zinc, selenium, and phytochemicals, both of which are associated with the prevention of chronic diseases [11]. Common examples of grains include rice, barley, wheat, millet, corn, sorghum, oats, and buckwheat [14].

### 3.1 Epidemiological evidence on the relationship between WG and GC

Given their central role in global diets [15], numerous studies have assessed grain consumption in relation to GC across Asian [16], [17], [18], [19], European [20], [21], [22], [23], [24], [25], [26] and American populations [27], [28], [29], [30], [31] (Table 2). A meta-analysis of 11 observational studies revealed that higher whole grain (WG) intake was associated with a lower risk of GC, while increased consumption of refined grains correlated with a higher risk; interestingly, total cereal intake did not show a significant association [32]. The authors highlighted that WG, which retains the germ, endosperm, and bran, provides fiber, vitamins, and minerals that may reduce inflammation and energy intake. In contrast, refined grains, primarily composed of endosperm, are quickly digested, potentially leading to glycemic overload, hyperinsulinemia, and elevated insulin-like growth factor I levels that can promote tumor growth [32].

**Table 2:** Characteristics and findings of studies on grains intake and gastric cancer risk.

Study	Country/publication year	Study design	Dietary assessment tool	Finding odd ratio: 95 % confidence interval (OR: 95 % CI) or hazard ratio: 95 % confidence interval (HR: 95 % CI)	Adjusted variables
Youssef et al. 18	Jordan, 2025	Case-control	Food frequency questionnaire	Highest vs. lowest white bread, OR = 3.13, CI: 1.57–6.21 rice Moderate vs. low OR = 0.38, CI: 0.18–0.81.	Age, sex, marital status, education level, previous BMI, smoking status and duration, family history of cancer, history of stomach ulcer and stomachache, physical activity (MET-min/week), and daily energy intake
Machida-Montani et al. 19	Japan, 2004	Case-control	Food frequency questionnaire	Highest vs. lowest Rice ≥ 5 cups/day vs. < 4 cups/day OR = 2.5, CI: 1.0–6.1	*H. pylori* infection, smoking status, Japan Agriculture membership, family history of gastric cancer, total vegetable intake, total fruit intake, salt intake, and total energy intake
Mathew et al. 20	India, 2000	Case-control	Interviewer administered food frequency questionnaire	Highest vs. lowest Rice OR = 3.9, CI: 1.6–10.1	Socio-demographic/economic background, tobacco chewing, tobacco smoking and alcohol habits
Wang et al. 21	China, 2012	Case-control	Structured questionnaire	Highest vs. lowest Cereals OR = 0.40, CI: 0.16–0.98	Educational status, smoking habit, alcohol consumption, and family history
La Vecchia et al. 22	Italy, 1988	Case-control	Structured questionnaire	Highest vs. lowest Whole grains OR = 0.40, CI: 0.16–0.98	Age, geographic site, sex, and education
Boeing et al. 23	Germany, 1991	Case-control	Interviewer administered questionnaire	Highest vs. lowest Whole grains OR = 0.37, CI:0.22–0.62	Age, sex, and hospital
Boeing et al.24	Poland, 1991	Case-control	Frequency questionnaire 43 food items	Highest vs. lowest Whole grains OR = 0.62, CI: 0.47–0.82	Age, sex, occupation, education, and residency
Jedrychowski et al. 25	Poland, 1992	Case-control	Structured questionnaire	Highest vs. lowest Whole grains OR = 0.18, CI: 0.07–0.44	Age, sex, education, occupation of the index person and residency
Hansson et al. 26	Sweden, 1993	Case-control	Structured questionnaire	Highest vs. lowest Whole grains OR = 0.89, CI: 0.79–1.01	Age, gender, socioeconomic status, and consumption of a food item during adolescence and 20 years prior to interview
Munoz et al. 27	Italy, 1997	Case-control	Structured questionnaire	Highest vs. lowest Whole grains OR = 0.63, CI: 0.28–1.40	Age, sex, area of residence and education
Chatenoud et al. 28	Italy, 199	Case-control	Structured questionnaire	Highest vs. lowest Whole grains OR = 0.50, CI: 0.40–0.70	Age and sex
Peres et al. 29	Brazil, 2022	Case-control	Food frequency questionnaire	São Paulo (Southeast region) Whole grain bread (>6.7 g/day) OR = 0.62, CI: 0.43–0.90 Pasta (>32 g/day) OR = 0.53, CI: 0.35–0.80 (Amazon region) Whole grain bread (>1.7 g/day) OR = 0.08, CI: 0.02–0.36 Pasta (>54 g/day) OR = 0.48, CI: 0.26–0.87	Age and sex
McCullough et al. 30	United State, 2001	Cohort	Food frequency questionnaire	Highes vs. lowest Whole grains > 4 vs. < 1 Sum of days/week Man HR = 0.90, CI: 0.77–1.06) Woman HR = 0.97, CI: 0.77–1.24	Age, education, smoking, BMI, multivitamin and vitamin C use, aspirin use, race, and family history
Wu-Williams et al. 31	United State, 1990	Case-control	Structured questionnaire	Highest vs. lowest Whole grains OR = 0.42, CI: 0.24–0.74	Age, sex, occupation, education, and residency
Kasum et al. 32	United State, 2002	Cohort	Food frequency questionnaire	6.9–12.5 vs. 13.0–108.5 Whole grains servings/week HR = 0.61, CI; 0.34–0.81	Age and energy intake
DeStefani et al.33	Uruguay, 2004	Case-control	Food frequency questionnaire	Highest vs. lowest Total grains OR = 1.83, CI: 1.17–2.85	Age, residence, urban/rural status, education, body mass index, tobacco smoking, alcohol drinking, and total energy intake

Recent evidence further supports the link between WG intake and a reduced risk of GC. In an umbrella review of 75 meta-analyses and systematic reviews, Kim et al. [33] concluded that higher WG consumption consistently offered protective benefits, while refined grain intake was associated with increased risk. This review included 11 studies on GC, showing that individuals with the highest WG intake had an inverse relationship with GC risk (RR = 0.64, 95 % CI: 0.53–0.79, *p* < 0.001) [11]. Similarly, a meta-analysis of 17 case-control and two cohort studies, encompassing 994,258 participants, found a 13 % reduction in GC risk associated with higher WG intake, which increased to 44 % at the highest levels of WG consumption. Conversely, refined grain intake was linked to a 36 % rise in GC risk, escalating to 63 % at elevated intake levels. Minimal consumption of refined grains showed no significant association with GC risk [14]. Subgroup analyses indicated variability based on refined grain type: rice intake was associated with a 53 % higher risk of GC, while non-rice refined grains correlated with a comparatively lower 28 % increase [14]. A recent systematic review and meta-analysis focusing on dietary components and GC risk in the Latin American population found no significant effect from cereals and tubers consumption on individuals with and without GC (OR 1.10; 95 % CI 0.82–1.47; *p* = 0.53) [34].

### 3.2 Protective mechanisms of WG against GC

WG are thought to protect against GC through various components and pathways. A significant aspect is their content of dietary fiber; it can eliminate nitrite in the stomach and lower the levels of nitroso compounds in highly acid conditions. An increased intake of nitrate may increase the risk of GC [35], 36]. A nationwide cohort study in Korea found that consuming at least 17.8 g/day of total dietary fiber was linked to a lower incidence of GC [37]. Additionally, a pooled analysis of 11 case-control studies from the Stomach Cancer Pooling (StoP) Project demonstrated a dose-response relationship for dietary fiber, with the highest quartile showing 28 % lower odds of developing GC compared to the lowest quartile [38]. Moreover, dietary fiber serves as a substrate for beneficial gut bacteria, stimulating the production of short-chain fatty acids such as butyrate, acetate, and propionate. For instance, butyrate functions as a histone deacetylase inhibitor, promoting the expression of anti-inflammatory and anti-carcinogenic genes in gastric tissues [39], 40]. Certain strains of *Lactobacillus* and *Bifidobacterium* possess probiotic properties that mitigate *Helicobacter pylori*-induced gastric inflammation *in vivo* by boosting mucosal immunity and decreasing oxidative stress [41]. These strains facilitate the production of anti-inflammatory cytokines, like IL-10 and TGF-β, while inhibiting pro-inflammatory cytokines, such as TNF-α [41]. Additionally, probiotics enhance the gastric mucosal barrier by increasing mucin secretion and maintaining epithelial integrity, which helps prevent microbial translocation and systemic inflammation [42].

In addition to dietary fiber, WG are rich in phenolic acids, particularly concentrated in their outer layers. Among polyphenols, gallic acid has been found to inhibit the growth of *H. pylori* cultures and the invasion and metastasis of cancer cells [43]. Similarly, Ho and colleagues [44] demonstrated that gallic acid can reduce the migration of human gastric carcinoma cells *in vitro* by inhibiting RhoB expression and modulating Akt signaling. Polyphenols also promote apoptosis: caffeic acid induces cell death by altering cellular Ca^2+^ homeostasis [45], ferulic acid activates caspase-3 and caspase-9 [42], and apigenin influences the expression of pro-apoptotic (Bax and caspase-3) and anti-apoptotic (Akt and Bad) proteins [46], 47]. Finally, polyphenols can modulate the activity of specific microRNAs: luteolin inhibits Bcl-2 expression by upregulating miR-34a, while p-coumaric acid exerts antitumor effects by regulating hsa-miR-30a-5p, hsa-miR-125a-5p, and hsa-miR-7-5p [48], [49], [50], [51], [52].

WG are also abundant in antioxidants, including vitamins, trace minerals, lignans, and phytoestrogens [53] that combat oxidative stress – a key factor in carcinogenesis driven by an imbalance of reactive oxygen species and antioxidant defenses [54]. Vitamin E, plentiful in WG, protects membrane polyunsaturated fatty acids from peroxidation, it inactivates or suppresses the production of oxygen free radicals and lipid peroxidation, which has major roles in carcinogenesis [55], scavenges the concentration of nitrite in the stomach, reduces, and inhibits nitrosation [56]. Selenium, another important antioxidant found in WG, acts as a cofactor for glutathione peroxidase and has been associated with reduced cell proliferation and suppression of carcinogen-induced neoplasia [53]. Its concentration varies with soil selenium levels, and refining processes significantly lower both vitamin E and selenium content. Since selenium levels in grains depend on local soil conditions, the same WG intake may deliver varying antioxidant levels across different regions, potentially explaining the differences in observed risk between countries [53]. Phytic acid, which is naturally present in WG, further enhances antioxidant activity by chelating metals and reducing iron-catalyzed redox reactions [55]. Treatment with phytic acid significantly suppressed the growth of the human gastric cancer cell line SGC-7901 and notably induced apoptosis through the downregulation of P53 protein expression [57].

**Figure 1:**
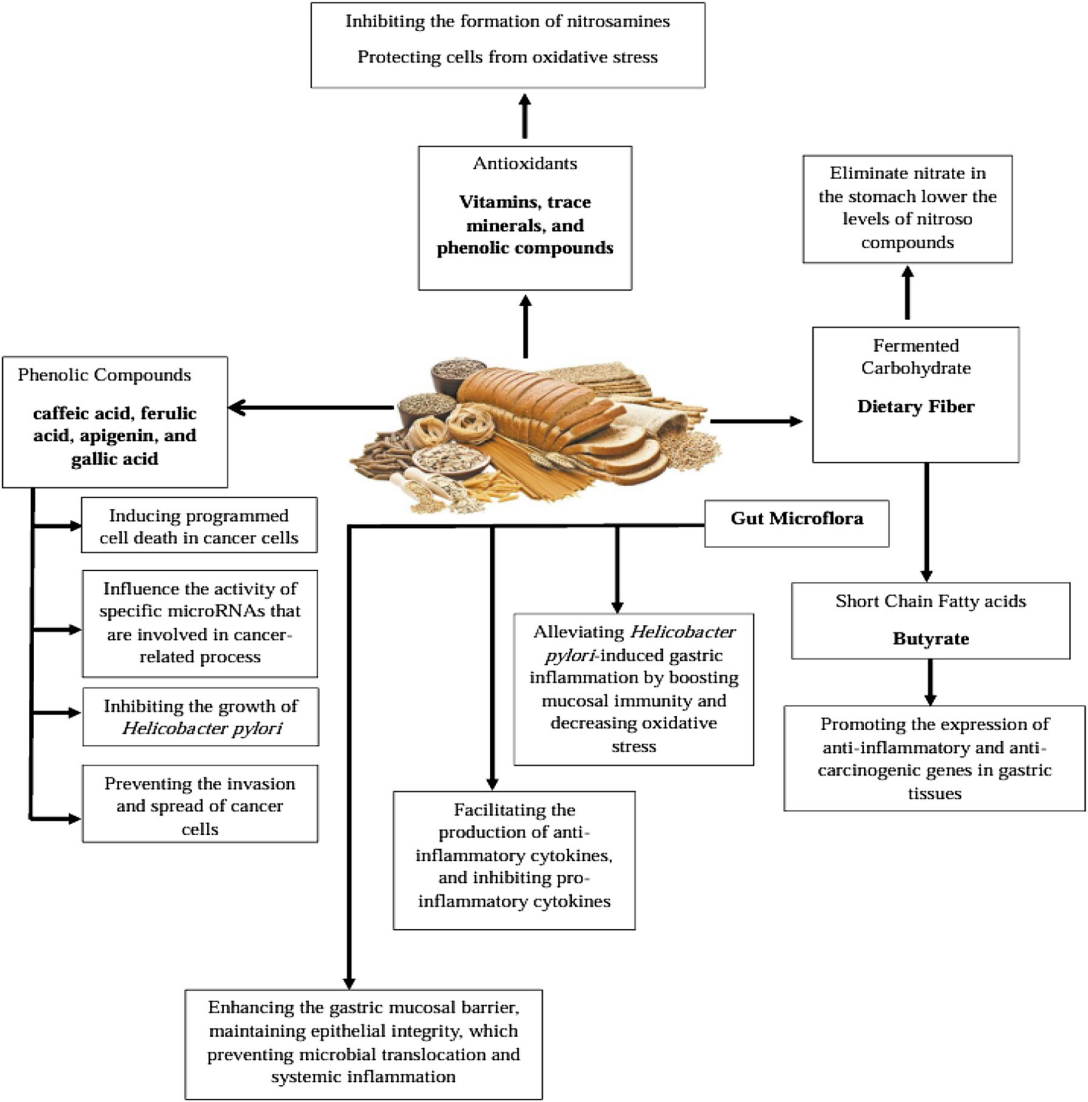
Biological mechanisms underlying the protective effects of whole grains against gastric cancer [35], 39], [41], [42], [43], [44], [54].

Whole grains may exert anticancer effects against gastric cancer through multiple complementary biological pathways. These include inhibition of nitrosamine formation, modulation of gut microbiota composition, and suppression of *H. pylori* growth. In addition, whole grains are rich in fiber, vitamins, minerals, and bioactive phytochemicals that contribute to reducing inflammation and oxidative stress – two central mechanisms implicated in gastric carcinogenesis. Collectively, these mechanisms may inhibit tumor initiation and progression while promoting cellular integrity and mucosal health (Figure 1) [35], 39], [41], [42], [43], [44], [54].

## 4 Legumes intake and GC

**Figure 2:**
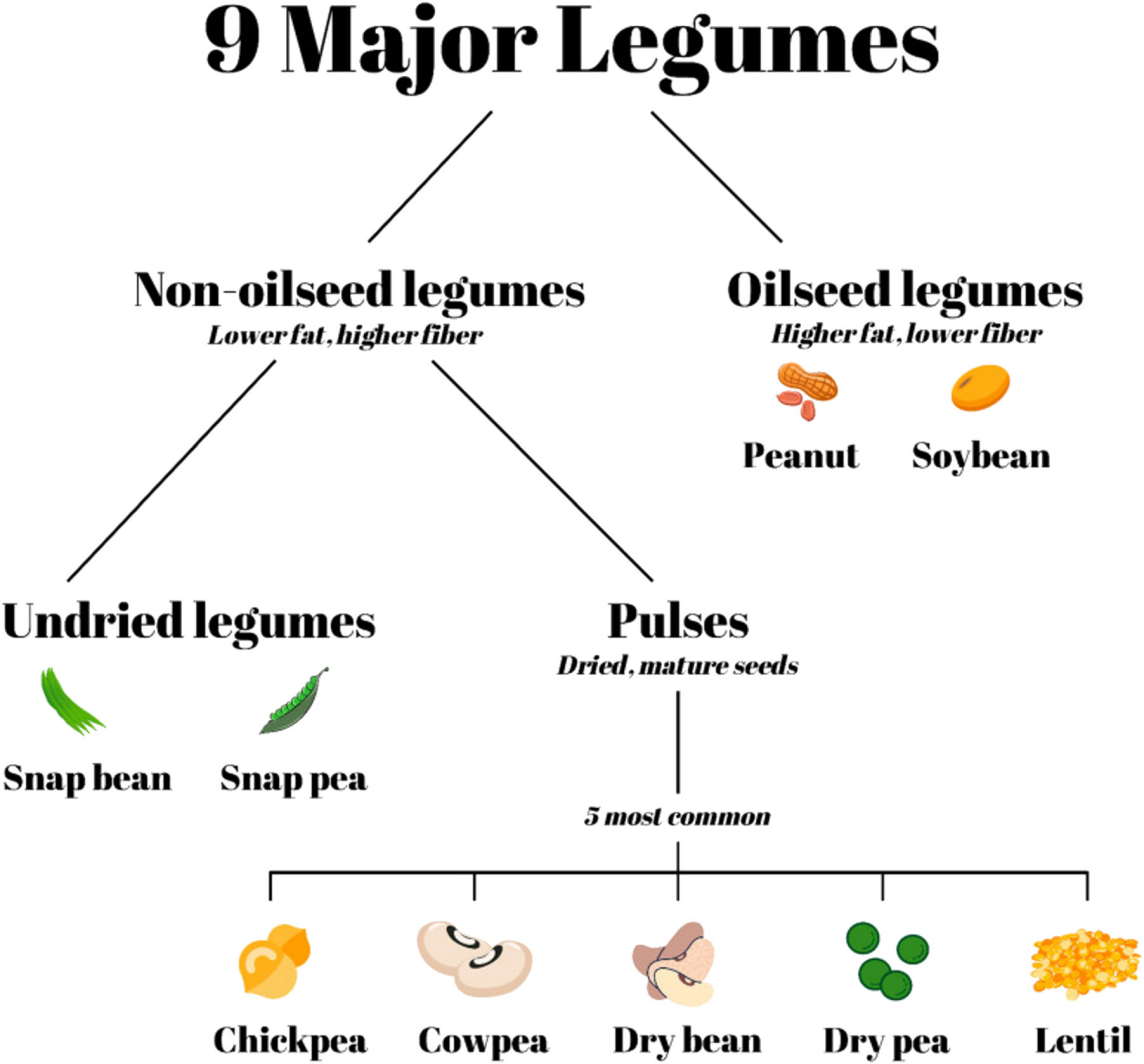
Legumes classification: Oilseeds versus non-oilseed [59].

Legumes are members of the *Fabaceae* family and encompass plants with edible parts such as pods, leaves, and stems. They serve as a nutritious staple across the globe, providing an affordable source of protein, vitamins, complex carbohydrates, and fiber [58]. According to Didinger and Thompson, legumes can be broadly categorized into oilseeds and non-oilseeds (Figure 2). Non-oilseeds are further divided into undried legumes and pulses. Pulses are dry seeds harvested as grains and do not include their green vegetable forms such as peas, chickpeas, and lentils – often referred to as grain legumes, pulses are distinct from undried legumes, which are collected before drying and include varieties such as snap beans and snap peas. Oil-producing legumes, particularly soybeans and peanuts, fall under the category of oilseed legumes [59].

### 4.1 Epidemiological evidence on the relationship between legumes and GC

Evidence regarding legume intake and GC risk is contradictory (Table 3). A case-control study conducted in Jordan found that hummus and cooked dried beans showed protective effects against GC risk. Conversely, the intake of green peas was surprisingly linked to a higher risk of GC [16]. In contrast, De Stefani et al. [60] reported no association between pulse consumption and GC in a case-control study from Uruguay in 2001. Additionally, De Stefani et al. [31] discovered that the highest consumption of pulses, compared to the lowest, was linked to a decreased risk of GC in another case-control study conducted in Uruguay in 2004.

**Table 3:** Characteristics and findings of studies on legumes intake and gastric cancer risk.

Study	Country/publication year	Study design	Dietary assessment tool	Findings Odd ratio: 95 % confidence interval (OR: 95 % CI) or hazard ratio: 95 % confidence interval (HR: 95 % CI)	Adjusted variables
Youssef et al. 18	Jordan, 2025	Case-control	Food frequency questionnaire	Moderate vs. lowe Hummus (chickpeas paste) OR = 0.48, CI: 0.26–0.97 Moderate vs. low Cooked dried beans OR = 0.40, CI: 0.20–0.81 Moderate vs. low Green peas OR = 2.19, CI: 1.24–3.88	Age, sex, marital status, education level, previous body mass index, smoking status and duration, family history of cancer, history of stomach ulcer and stomachache, physical activity (MET-min/week), and daily energy intake
Machida-Montani et al. 19	Japan, 2004	Case-control	Food frequency questionnaire	Moderate vs. low Miso soups 3 cups/day vs. < 3 cups/day OR = 1.8, CI: 1.0–3.3	*H. pylori* infection, smoking status, Japan Agriculture membership, family history of gastric cancer, total vegetable intake, total fruit intake, salt intake, and total energy intake
DeStefani et al.33	Uruguay, 2004	Case-control	Food frequency questionnaire	Highest vs. lowest Pluses OR = 0.56, CI:0.37–0.85	Age, residence, urban/rural status, education, body mass index, tobacco smoking, alcohol drinking, and total energy intake
DeStefani et al.63	Uruguay, 2001	Case-control	Food frequency questionnaire	Highest vs. lowest Legumes +10.8 g/day vs. ≤ 1.1 g/day OR = 0.62, CI: 0.33–1.19	Age, residence, urban/rural status, education, body mass index, total energy, tobacco smoking and alcohol drinking.
Nam et al. 65	Korea, 2024	Case-control	Interviewer administered questionnaire	Highest vs. Lowest soybean paste stew ≥5 times/week vs. 0 time/week Women (OR = 7.58, CI: 3.20–17.99) Men (OR = 3.03, CI: 1.61–5.88)	Age, sex, BMI, smoking status, diabetes, hypertension, cerebrovascular disease, physical activity, family history of GC, and occupation – were adjusted for in the multivariable analysis. Model II included adjustments for *H*elicobacter *pylori* IgG
Shin et al. 71	Korea, 2023	Cohort	Self-administered questionnaire	soybean paste ≥2 times/week vs. 0 HR = 0.63, CI: 0.45–0.89	Age, sex, smoking, body mass index, educational level, family history of GC, total energy intake, and alcohol consumption
Nieuwenhuis and Brandt, 75	Netherlands, 2018	Cohort	Self-administered questionnaire	Highest vs. Lowest Peanuts +10 g/day vs. 0 g/day Gastric cardia adenocarcinoma HR = 1.0, CI: 0.62–1.61 Gastric non- cardia adenocarcinoma HR = 0.84, CI: 0.63–1.13 Peanut butter +5 g/day vs. 0 g/day Gastric cardia adenocarcinoma HR = 0.78, CI: 0.44–1.37 Gastric non- cardia adenocarcinoma HR = 0.88, CI: 0.63–1.21	Age, sex, cigarette smoking, frequency, and duration, body mass index, educational level, family history of GC, total energy intake, and alcohol consumption

A large Italian-Swiss case-control network also found no significant association between total legume intake and GC, although legumes appeared to be protective against colorectal cancer [61]. In Korea, a multicenter, case-control study revealed that non-fermented soy products (e.g., tofu) were associated with a lower risk of GC. Conversely, high-salt fermented soybean dishes exhibited a V/U-shaped relationship with increased risk, particularly among women and those with a preference for salty foods [62]. Furthermore, Machida-Montaniet al. [17] demonstrated that moderate consumption of miso soup, made from soybean paste, was positively associated with GC as compared to low consumption levels.

These findings suggest that the method of preparation plays a critical role. Non-fermented soy foods are typically low in sodium and served plainly, while fermented pastes and pickles are salt-heavy and consumed in small, concentrated portions, which may lead to differing associations within “soy.” Salt has detrimental effects on the gastric mucosa by increasing the levels of gastric carcinogens, such as N-methyl-N-nitro-N-nitrosoguanidine. It promotes gastric epithelial hyperplasia and loss of parietal cells while also enhancing the colonization of *H. pylori* in mice [63], 64]. A systematic review and meta-analysis of case-control studies found a significantly positive association between high salt intake and GC versus low salt intake (OR = 1.55, 95 % CI (1.45, 1.64); *p* < 0.001) [65]. Additionally, results can vary based on cultural eating patterns – whether legumes are part of a plant-forward meal (e.g., a stew with vegetables and olive oil) or are paired with saltier, meat-heavy dishes.

This variability helps explain why a meta-analysis of 52 observational studies on overall cancer risk, including gastric cancer, found protective associations for total soy products, tofu, and soymilk, but not for fermented soy products [66]. Supporting this observation, an Italian case-control study identified an inverse association between GC risk and dietary isoflavone intake, predominantly derived from non-soy legumes [67].

Oilseed legumes, such as soybeans and peanuts have also been the subject of research. A 2023 prospective cohort study in Korea found that increased consumption of soybean products was linked to a lower risk of GC [68]. Similarly, a systematic review indicated that overall soybean intake was associated with a 36 % decrease in GC incidence, primarily due to non-fermented soybean products; in contrast, a high intake of fermented soybean products was linked to an increased risk [69]. Additionally, a separate meta-analysis revealed an inverse relationship between soy consumption and the risk of gastrointestinal cancers, including GC [70]. Another prospective cohort study found that higher intake of nuts and peanut butter was inversely related to GC risk in a dose-responsive manner [71]. In contrast, a Dutch prospective cohort study showed that the greatest peanut and peanut butter consumption was not significantly associated with a lower risk of GC [72].

### 4.2 Protective mechanisms of legumes against GC

**Figure 3:**
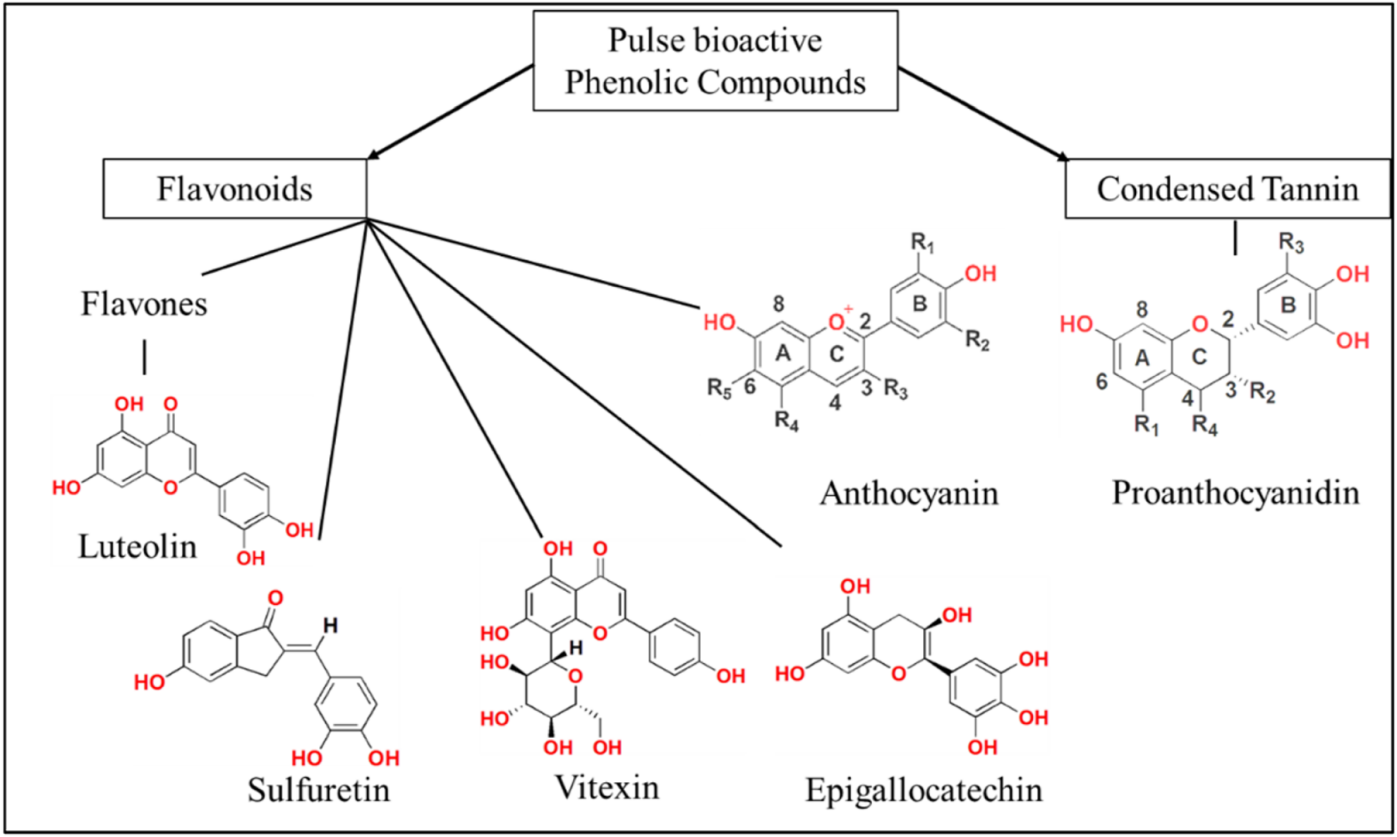
Bioactive compounds in pulses: Flavonoids, anthocyanins, and tannins [8].

Pulses, such as dried beans and lentils, consist of three primary seed structures: the endosperm, seed coat, and embryo. The endosperm serves as a storage site for carbohydrates, proteins, and fats, while the seed coat is notably high in polyphenols (see Figure 3) [73]. Polyphenols possess various anticarcinogenic properties, including antioxidant [74] and anti-inflammatory effects [75]. Some have also demonstrated antimicrobial activity, inhibiting the growth of *H. pylori in vitro* [76]. Additionally, flavonoids engage with numerous molecules involved in apoptosis and cell proliferation pathways, influencing their expression or activity [77]. Compounds such as genistein and kaempferol influence essential signaling pathways, including PI3K/Akt, MAPK, and NF-kB, which play roles in cell growth, survival, and inflammation [78], 79]. Furthermore, these polyphenols can encourage apoptosis and restrict angiogenesis, ultimately hindering tumor growth and metastasis [80]. The genistein may suppress NF-κB signaling associated with inflammation and GC [81], and reduce growth and proliferation of stomach cancer cells [82].

Moreover, research has shown that flavonoids exert their anti-tumor effects by inhibiting tumor growth, proliferation, invasion, and metastasis, while also promoting cell death through several mechanisms, including apoptosis, autophagy, ferroptosis, and pyroptosis [83]. Quercetin, a flavonol, has been shown to inhibit GC cell proliferation and disrupt cell-cycle progression [84].

Legumes also offer bioactive proteins and lipids. Lectins can attach to carbohydrate residues on tumor cells, potentially inhibiting growth, activating immune responses, and inducing apoptosis. Protease inhibitors, such as Bowman–Birk inhibitors (BBI), may block enzymes necessary for tumor progression. While pulses are low in fat, they contain significant fatty acids like oleic and linoleic acids. Additionally, components of legumes can facilitate the production of beneficial metabolites, such as butyrate, through gut microbial fermentation [73].

The method of preparation can influence these effects: soaking, sprouting, and thorough cooking can reduce antinutrients (such as phytates and active lectins) and enhance digestibility. Fermentation can convert isoflavones into more bioavailable forms, although it often increases sodium content. These processes may contribute to anti-inflammatory and anticancer effects by modulating gut microbiota and alleviating chronic inflammation, which is a major factor in carcinogenesis. Together, these components can affect gene expression pathways related to tumor growth in the gastrointestinal tract. In dietary practice, meals rich in legumes, particularly when prepared with minimal salt, can help reduce nitrosative stress and, when replacing red or processed meats, decrease exposure to heme and nitrites associated with gastric carcinogenesis.

## 5 The combined effect of WGs and legumes consumption on GC risk

Whole grains (WG) and legumes may be linked to a lower incidence of gastric cancer (GC). Although their combined effect as a distinct exposure has not been extensively studied, both foods have been individually examined within dietary patterns, particularly the Mediterranean and DASH diets. These diets emphasize fruits, vegetables, whole grains, legumes, seeds, nuts, and olive oil, while being lower in saturated fats, sodium, alcohol, and processed meats compared to typical Western diets [85]. Multiple studies have indicated that adherence to the Mediterranean and DASH dietary patterns correlates with a reduced cancer risk, likely due to their anti-inflammatory and antioxidant properties that impact tumor-related pathways associated with gastric carcinogenesis [8], 82], 86], 87].

However, the observed associations cannot be attributed solely to whole grains and legumes. Other protective components, particularly fruits, vegetables, and unsaturated fats, may confound or mediate this relationship, complicating efforts to isolate the specific contribution of combined WG and legume intake [9]. A hospital-based multi-case-control study conducted in Spain by Oncina-Canovas et al. [88] examined three pro-vegetarian dietary patterns in relation to cancers of the esophagus, stomach, and pancreas. They found that general and healthful pro-vegetarian patterns, which included legumes, were associated with a lower risk of GC.

In summary, current evidence supports plant-forward dietary patterns that include both whole grains and legumes, but direct assessments of their joint effects are limited. Future studies should clearly define how they will measure the combined intake of whole grains and legumes, use consistent intake units across participants and studies, and statistically control for other dietary components. This approach will help determine whether any risk reduction from consuming both foods is merely the sum of their individual effects or if it indicates additional synergistic benefits.

## 6 Limitations and research gaps

The limitations of this narrative review are variable. First of all, the selection bias of studies that have been examined and the lack of quality appraisal. Secondly, it is totally based on observational studies, which depend on self-reported dietary data that can introduce bias and inaccuracies. Thirdly, confounding factors such as socioeconomic status, lifestyle choices, and other dietary habits may not be adequately controlled, thereby affecting the validity of the findings [89]. The associations of WG and legume consumption and risk of GC are inconclusive. Fourthly, comparing studies on whole grains and legumes presents challenges due to varying definitions, serving sizes, and dietary assessment tools. Many studies inconsistently combine or separate grain and legume categories. There is also a lack of consistency in how legumes are prepared – whether fermented or non-fermented, salted or unsalted, soaked or sprouted, lightly cooked or long-simmered/pressure-cooked. Cultural practices vary significantly; in some settings, grains and legumes are consumed in vegetable-rich stews, while in others, they are used as salty condiments alongside meat. This variation affects sodium intake, accompanying foods, and overall diet quality. Furthermore, results can fluctuate based on population characteristics and overall diet quality, complicating the isolation of true effects. To establish clearer evidence, shared definitions and portion metrics (grams per day) are essential, along with consistent reporting of grain and legume categories, repeated dietary assessments, and large prospective or pooled analyses that effectively control for confounding variables and explicitly test dose–response relationships.

To enhance comparability, future research should specify the types and preparation methods of whole grains and legumes, standardize intake metrics, capture sodium and fermentation details, and employ well-powered prospective or pooled designs that investigate dose–response relationships and key effect modifiers while carefully adjusting for overall dietary patterns.

## 7 Conclusions

This narrative review suggests that WG and legumes may offer protection against GC. Substituting refined grains with whole grains provides dietary fiber, vitamins, minerals, and phytochemicals that promote a healthy gut microbiome while reducing inflammation and oxidative stress, potentially lowering GC risk. The review also highlights legumes – both oilseed and non-oilseed – emphasizing pulses for their fiber and antioxidant content, as well as their polyphenols and isoflavones, which may help limit tumor growth and inflammation. While plant-forward dietary patterns offer a broader context, the joint effect of WG and legumes remains unclear. Future research should investigate dose–response relationships, consider legume types and preparation methods, and isolate the effects of whole grains and legumes from other dietary factors through randomized controlled trials, microbiome-focused studies, and multicenter longitudinal cohorts. Well-designed prospective studies and pooled analyses should explicitly assess combined WG and legume intake to determine whether any protective effect is additive or synergistic.
